# Peripheral airway dysfunction in prematurity‐associated obstructive lung disease identified by oscillometry

**DOI:** 10.1002/ppul.26658

**Published:** 2023-09-13

**Authors:** Michael Cousins, Kylie Hart, Bence L. Radics, A John Henderson, Zoltán Hantos, Peter D. Sly, Sailesh Kotecha

**Affiliations:** ^1^ Department of Child Health Cardiff University School of Medicine Cardiff UK; ^2^ Department of Paediatrics Cardiff and Vale University Health Board Cardiff UK; ^3^ Department of Pathology University of Szeged Szeged Hungary; ^4^ MRC Integrative Epidemiology Unit, Population Health Sciences, Bristol Medical School University of Bristol Bristol UK; ^5^ Department of Anesthesiology and Intensive Therapy Semmelweis University Budapest Hungary; ^6^ Child Health Research Center The University of Queensland South Brisbane Australia

**Keywords:** airway obstruction, albuterol, oscillometry, premature birth, respiratory mechanics

## Abstract

**Introduction:**

Mechanisms underlying lung dysfunction after preterm birth are poorly understood. Studying phenotypes of prematurity‐associated lung disease may aid understanding of underlying mechanisms. Preterm‐born children with and without lung dysfunction and term controls were assessed using oscillometry before and after exercise, and after postexercise bronchodilation.

**Methods:**

Preterm‐born children, born at gestation of 34 weeks or less, were classified into those with prematurity‐associated obstructive lung disease (POLD; FEV_1_ < LLN, FEV_1_/FVC < LLN), prematurity‐associated preserved ratio of impaired spirometry (pPRISm; FEV_1_ < LLN, FEV_1_/FVC ≥ LLN) and compared to preterm (FEV_1_ ≥ LLN) and term controls (%predicted FEV_1_ > 90%). All children underwent cardiopulmonary exercise, and oscillometry assessment at baseline, postexercise, and after postexercise bronchodilator administration.

**Results:**

From 241 participants aged 7–12 years, complete data were available from 179: 15 children with POLD and 11 with pPRISm were compared with 93 preterm and 60 term controls. POLD group, when compared to both control groups, had impaired impedance, greater resistance, more negative (greater magnitude) reactance at low frequencies, and also had decreased compliance. pPRISm group demonstrated impaired reactance and compliance compared to term controls. No differences were noted between the preterm and term controls. Exercise had little impact on oscillometry values, but children with POLD had greatest improvements after postexercise bronchodilator administration, with decreased resistance and decreased magnitude of reactance, particularly at low frequencies.

**Conclusion:**

Preterm‐born children with obstructive airway disease had the greatest oscillometry impairments and the largest improvements after postexercise bronchodilator compared to control groups. Oscillometry can potentially be used to identify preterm‐born children with lung disease to institute treatment.

## 1 INTRODUCTION

Preterm birth disrupts the normal in utero development of lung tissue and pulmonary vasculature resulting in longer term increase in respiratory symptoms, deficits of lung function and increased in hospitalization.^1^ Most focus thus far has been on preterm children born at less than 32 weeks’ gestation including those who developed chronic lung disease of prematurity (CLD, also known as bronchopulmonary dysplasia or BPD) in infancy. However, respiratory disease is not limited to these groups as those born late preterm especially at 33–34 weeks’ gestation at birth are also at risk of respiratory symptoms and decreased lung function.^2^, ^3^, ^4^ While there are many studies reporting the impact of preterm birth on respiratory symptoms,^5^, ^6^, ^7^ health care access,^8^ lung function,^1^ and exercise outcomes,^9^ less is known about the impact of prematurity‐associated lung disease on respiratory mechanics. Oscillometry (also known as Forced Oscillation Technique or FOT), employs the technique of superimposing small amplitude oscillations onto tidal breathing to determine the impedance of the respiratory system (Z_rs_).

Oscillometry is a particularly useful tool in pediatric populations as it only requires tidal breathing, and not complicated breathing manoeuvres such as forced expiration in spirometry. Oscillometry has been used to evaluate respiratory mechanics in preterm populations, from infancy, preschool ages, and children up to adulthood; thus far, mostly focusing on children who had CLD in infancy. Children with CLD have lower reactance values (i.e., likely impaired compliance) compared to preterm children without CLD and term counterparts at preschool ages and in older children,^10^, ^11^ with decline in respiratory reactance observed from early to mid‐childhood in very preterm survivors.^12^ Reactance appears particularly sensitive in identifying respiratory disease in preterm‐born children.^13^ However, limited data are available for effects of bronchodilators, especially after exercise, on respiratory mechanics in preterm children. Absolute and relative changes without adjustment for some resistance parameters have been reported in one study when compared to term controls. However, these differences largely disappeared after adjustment for baseline lung function.^14^ Respiratory mechanics by oscillometry following exercise and after postexercise bronchodilator in preterm populations have yet to be evaluated.

Additionally, preterm‐born children with evidence of lung disease are unlikely to fall within a single phenotype.^15^ While obstructive airway disease has been described for the preterm population,^11^ other phenotypes are less well reported.^16^ The concept of preserved ratio of impaired spirometry (PRISm, low FEV_1_ < LLN, FEV_1_/FVC ≥ LLN)^17^, ^18^ has been described in middle‐ to old‐aged adults but is less well described in children. This is an important concept as PRISm is associated with longer term poor cardiorespiratory outcomes and with all‐cause mortality in adult populations.^17^, ^18^ We have recently described the prematurity‐associated PRISm (pPRISm) phenotype^19^ and also showed that this phenotype is associated with impaired exercise capacity with reduced O_2_ uptake at peak exercise using a cycle ergometer, with little postexercise bronchodilator response.^20^ Along with low functional residual and total lung capacities, this perhaps suggests a fixed structural lung disease in a pediatric preterm PRISm population that may have significant long‐term impact given the outcomes identified from adult studies.^20^

Oscillometry is likely to provide significant information about how the airways and lung tissues may be affected after preterm birth, especially during exercise when dynamic changes of the respiratory mechanics are occurring. We hypothesized that differences in respiratory mechanics using oscillometry can be demonstrated at baseline, after maximal exercise and after postexercise bronchodilator when preterm‐born children with different spirometric phenotypes are compared with term‐born controls.

## 2 METHODS

### 2.1 Population

Children born preterm and at term between 2005 and 2011 were prospectively recruited as part of the Respiratory Health Outcomes in Neonates (RHiNO) study (EudraCT: 2015‐003712‐20) as described in more detail in the online supplement.^21^, ^22^ These children were identified during a previous questionnaire study,^2^, ^6^ and were recruited to participate in the current study, which ran between January 2017 and August 2019. Inclusion criteria were gestational age at birth ≤34 weeks gestation for preterm‐born children and at ≥37 weeks gestation for term‐born children, age 7–12 years, and geographically accessible. Children with significant congenital, cardiac, or neurodevelopmental abnormalities were excluded. Recruitment was postponed in children with a recent (within the past 3 weeks) respiratory tract infection.

#### 2.1.1 Spirometry and cardiopulmonary exercise testing (CPET)

Spirometry and exercise testing are described in more detail in the online supplement. Spirometry was performed according to the ATS/ERS guidelines^23^ using the MasterScreen Body and PFT systems with SentrySuite measurement software version 2.17 (Vyaire Medical). Spirometry measures were referenced against the Global Lung Initiative (GLI) equations.^24^ CPET was performed on a Pediatric Cycle Ergometer (Lode) linked to a Masterscreen CPX system (Vyaire Medical). A test was deemed to be “maximal” if it met two or more of the following criteria: respiratory exchange ratio >1.00; heart rate ≥80% predicted (220 beats per minute − age in years); ≥9/10 on OMNI scale (pictorial scale for rating of perceived exertion^25^); oxygen uptake plateau based on visual analysis.

#### 2.1.2 Spirometry phenotypes

Lower limit of spirometry measures (defined as z‐score < −1.64 based on GLI equations)^24^ were used to classify the children into three preterm phenotypes of interest as below:

∘ Prematurity‐associated obstructive lung disease (POLD): FEV_1_ < LLN, FEV_1_/FVC < LLN;
∘ Prematurity‐associated preserved ratio of impaired spirometry (pPRISm): FEV_1_ < LLN, FEV_1_/FVC ≥ LLN;
∘ Preterm controls (PT_c_): FEV_1_ ≥ LLN.

Term‐born children with a priori defined percent predicted (%)FEV_1_ > 90% were recruited as term controls (T_c_). As term children with %FEV_1_ ≤ 90% predicted were not recruited, the term control group was not able to include children with %FEV1 between LLN and 90%. These phenotypes and definitions are summarized in Table SE1 in the online Supporting Information.

### 2.2 Oscillometry testing

Oscillometry testing was performed using a custom‐built loudspeaker‐in‐box device, designed to operate during postexercise rapid breathing (see details in the online Supporting Information). Zrs was measured in the 4–32‐Hz range.^26^, ^27^ The test was performed with the child sitting upright and connected to the system via a Microgard‐II microbial filter (Vyaire). A nose‐clip was worn during testing and the children (or their parents or research staff where necessary) firmly held their cheeks with their fingers and palms of hands to stop any soft tissue vibrations. A loudspeaker then generated soundwaves at even frequencies between 4 and 32 Hz. Impedance was measured at the mouth using the pressure and flow sensors, for each individual frequency. Results from the individual frequencies were displayed in the form of a spectrum. A minimum of three recordings were obtained, and artefact‐free segments of at least 16 s were selected, analysed, and averaged, allowing calculation of the mean impedance at each individual frequency. Recordings for data analyses were selected after visual inspection.

Resistance (Rrs) and reactance (Xrs) at 6 and 20 Hz were analysed. In addition, the averaged resistance across all recorded frequencies between 6 and 20 Hz was calculated (Rrs_mean_). A frequency dependence of Rrs was characterized as the difference between resistance at 6 Hz (Rrs_6_) and 20 Hz (Rrs_20_) (Rrs_6–20_). Compliance (Crs) was calculated by fitting an inertance‐compliance model to the Xrs data^26^, ^27^; resonance frequency (*ƒ*res) was estimated from this model fitting as the frequency where the reactance equalled 0. The area enclosed between zero and Xrs between 6 Hz and *f*res (AX) was also calculated.^28^ Using reference equations derived from healthy children from Australia and Italy,^29^ Rrs_6_, Xrs_6_, and AX were converted to z‐scores after data transformation (natural logarithm Rrs_6_; square root of absolute value of (Xrs_6_‐ 10); square root of AX. Z‐scores were calculated as the measured minus predicted value divided by the standard error of the estimate noted in the Calogero publication.^29^

Oscillometry was performed at three time points: at baseline (before spirometry), 20 min following maximal exercise testing (between serial spirometry measurements) and 15 min after administration of postexercise bronchodilator (400 μg of salbutamol [Salamol^@^, TEVA UK Limited] administered with an MDI using a Volumatic spacer [GSK]) (following spirometry), as described in the online Supporting Information. This was performed the same for all participants. A positive bronchodilator response was classed as a greater than 40% increase in Rrs_6_, greater than negative 50% increase in Xrs_6_ and greater than 80% increase in AX.^30^

### 2.3 Ethical approval

Ethics approval for the RHiNO study was granted by the Southwest Central Bristol Ethics Committee (Ref 15/SW/0289). Parents and children provided informed written consent and assent, respectively.

### 2.4 Statistical analysis

Multiple group comparisons and differences between groups of continuous data were performed using one‐way analysis of variance (ANOVA) with Bonferroni correction. Categorical data were assessed using Pearson's *χ* ^2^ tests. Within‐group and between group comparisons across time points were measured with two‐way repeat measures ANOVA, with Bonferroni correction. Children were included in analysis if they had oscillometry data at all three times points of testing; therefore, participants with missing data at one or more time points (due to equipment or recording issue, time constraint, test quality, or declining test) were excluded from repeated measures analysis. Statistical analysis was performed using SPSS version 26 (IBM).

## 3 RESULTS

### 3.1 Participants

**Figure 1:**
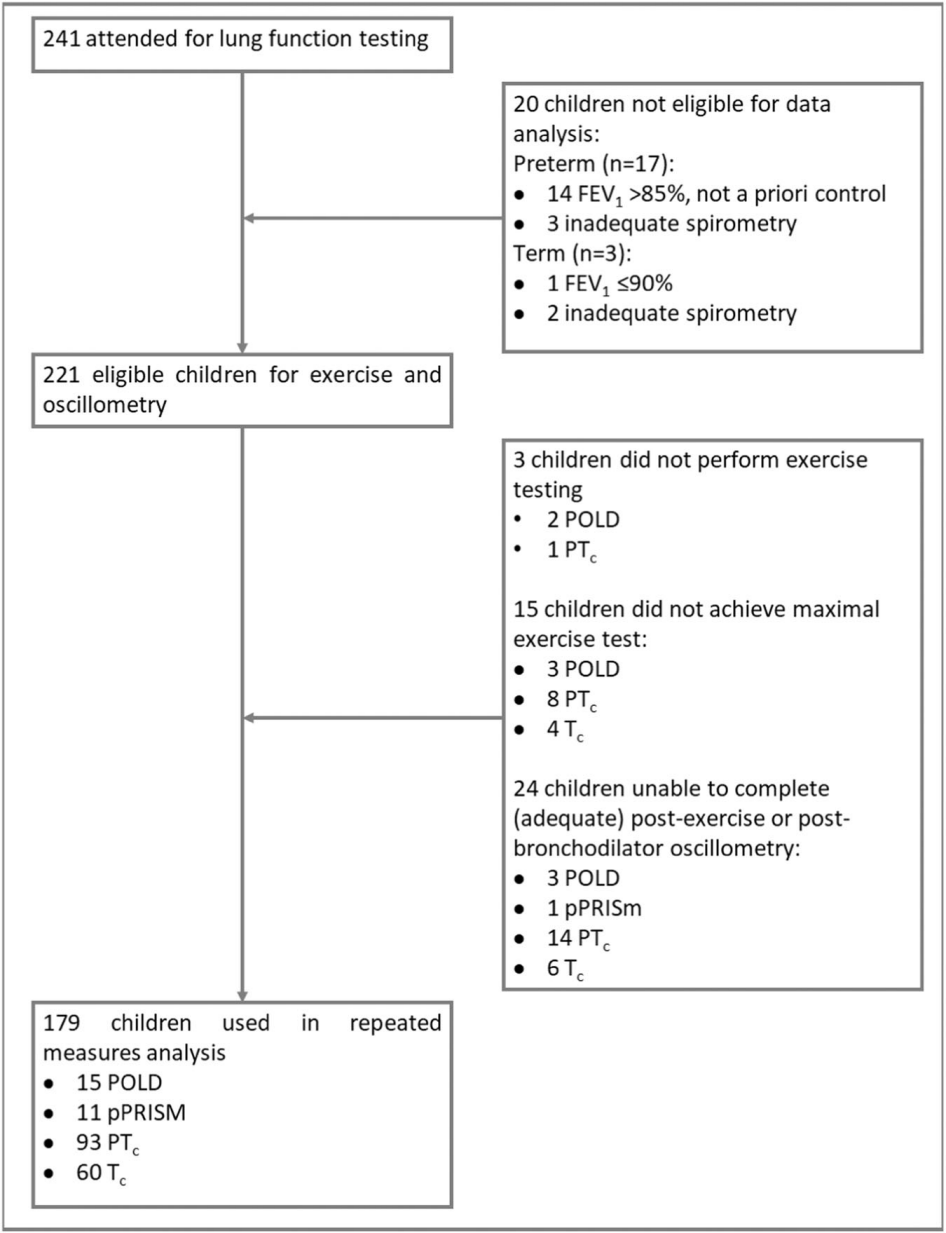
Flow diagram outlining recruitment numbers for lung function testing and numbers for those included in final analysis.

From 241 participants attending, 20 were excluded after spirometry (see Figure 1). In addition, 3 children did not perform exercise testing (2 due to time constraint and 1 being unable to perform the test) and 15 children did not achieve maximal exercise testing, as per our criteria. Of those not achieving maximal exercise testing, a slightly greater number were from those children with POLD (13% vs. 7% of preterm controls and 6% of term controls). A total of 24 of the remaining 203 children had one or more time points missing from their oscillometry testing for a variety of reasons [missed or declined test largely due to time constraint (*n* = 15), recording issue (*n* = 6), and suboptimal quality of test (*n* = 3)]. Thus, 179 children were included in repeated measures analysis of oscillometry data and were classified into: 15 with POLD, 11 pPRISm, 93 PT_c_, and 60 T_c_ using their spirometry results.

### 3.2 Participant characteristics

Participant characteristics are summarized in Table 1. Anthropometric measurements at the time of testing were similar between groups; no difference in height was noted between the groups; however, PT_c_ children were slightly older than their term counterparts. Children in the POLD group were born earlier and had lower birth weight than preterm controls (29.1 vs. 31.1 weeks’ gestation and 1307 vs. 1710 g, respectively). There were no differences for invasive ventilation or for CLD rates between the preterm groups. The POLD group had higher rates compared to term controls for wheeze ever (87% vs. 23%; and vs. 47% in PT_c_), recent (12 months) wheeze (47% vs. 13%), asthma diagnosis (40% vs. 8%), salbutamol use (33% vs. 7%) and current maternal smoking (13% vs. 0%).

**Table 1:** Baseline characteristics of participants including anthropometric, perinatal and respiratory details for preterm obstructive lung disease (POLD), preterm preserved ratio impaired spirometry (pPRISm), preterm (PTc), and term (Tc) controls.

	POLD (*n* = 15)	pPRISm (*n* = 11)	PT_c (_ *n* = 93)	T_c_ (*n* = 60)
**Current demographics**				
Age, years	10.9 (10.1 to 11.6)	11.1 (10.2 to 12.1)	**11.1** (**10.9 to 11.4)^₴₴^**	10.4 (10.1 to 10.6)
Male, *n* (%)	9 (60%)	2 (18%)	47 (51%)	31 (52%)
Height, cm	141.1 (135 to 147.2)	142.8 (133.3 to 152.3)	146.4 (144.5 to 148.4)	142.6 (140.3 to 145)
Height, Z‐score	−0.28 (−0.89 to 0.33)	−0.32 (−1.35 to 0.71)	0.28 (0.11 to 0.46)	0.38 (0.12 to 0.63)
Weight, kg	36.7 (31.1 to 42.3)	35.5 (26.5 to 44.6)	39.9 (37.7 to 42)	36.2 (34.2 to 38.2)
Weight, Z‐score	0.01 (−0.71 to 0.74)	−**0.63** (−**1.84 to 0.58)** ^¥¶^	0.34 (0.13 to 0.56)	0.35 (0.11 to 0.59)
BMI, kg/m^2^	18.1 (16.3 to 20)	16.8 (14.4 to 19.2)	18.4 (17.6 to 19.1)	17.6 (17 to 18.2)
BMI, Z‐score	0.21 (−0.52 to 0.95)	−0.67 (−1.74 to 0.39)	0.20 (−0.07 to 0.46)	0.22 (−0.04 to 0.47)
**Perinatal demographics**				
Gestation, decimal weeks	**29.1** (**27.4 to 30.7)** ^**† ‡‡‡**^	**30** (**28 to 32)^¶¶¶^**	**31.1** (**30.6 to 31.7)^₴₴₴^**	40 (39.7 to 40.3)
Birth weight, grams	**1307** (**1008 to 1606)** ^**† ‡‡‡**^	**1487** (**1077 to 1898)^¶¶¶^**	**1710** (**1594 to 1826)^₴₴₴^**	3495 (3375 to 3615)
Birth weight, Z‐score	−0.21 (−0.79 to 0.38)	0.03 (−0.73 to 0.78)	0.17 (−0.11 to 0.46)	0.04 (−0.19 to 0.26)
IUGR, *n* (%)	2 (13%)	2 (18%)	16 (17%)	3 (5%)
Antenatal steroids, *n* (%)	**13** (**87%)^‡‡‡^**	**10** (**91%)^¶¶¶^**	**77** (**83%)^₴₴₴^**	0 (0%)
Invasive ventilation, *n* (%)	**10** (**67%)^‡‡‡^**	**4** (**36%)^¶¶¶^**	**35** (**38%)^₴₴₴^**	0 (0%)
CLD, *n* (%)	**6** (**40%)^‡‡‡^**	**3** (**27%)^¶¶¶^**	**19** (**20%)^₴₴₴^**	0 (0%)
**Respiratory history**				
Doctor‐diagnosed asthma, *n* (%)	6 (40%)^**‡**^	2 (18%)	22 (24%)	5 (8%)
Wheeze ever, *n* (%)	**13** (**87%)** ^**† ‡‡‡**^	6 (55%)	**44** (**47%)** ^₴^	14 (23%)
Recent wheeze, *n* (%)	**7** (**47%)** ^‡^	2 (18%)	22 (24%)	8 (13%)
Current salbutamol use, *n* (%)	**5** (**33%)** ^‡^	1 (9%)	19 (20%)	4 (7%)
Current maternal smoking, *n* (%)	**2** (**13%)** ^‡^	1 (9%)	6 (7%)	0 (0%)

*Note*: Results expressed as mean and 95% confidence intervals for continuous data (one‐way ANOVA with Bonferroni correction) or number and % proportion (Pearson's *χ* ^2^ test) unless otherwise specified.

Significance symbols: *POLD vs. pPRISm, ^†^POLD vs. PTc, ^‡^POLD vs. Tc, ^¥^pPRISm vs. PTc, ^¶^PRISm vs. Tc, ^₴^PTc vs Tc.

(“Single symbol” denotes significance level <0.05, “double symbol” <0.01, “triple symbol” <0.001. Bold values indicate statistical significance between groups.)

Abbreviations: ANOVA, analysis of variance; BMI, body mass index; CLD, chronic lung disease of prematurity; IUGR, intrauterine growth restriction.

### 3.3 Oscillometry

**Figure 2:**
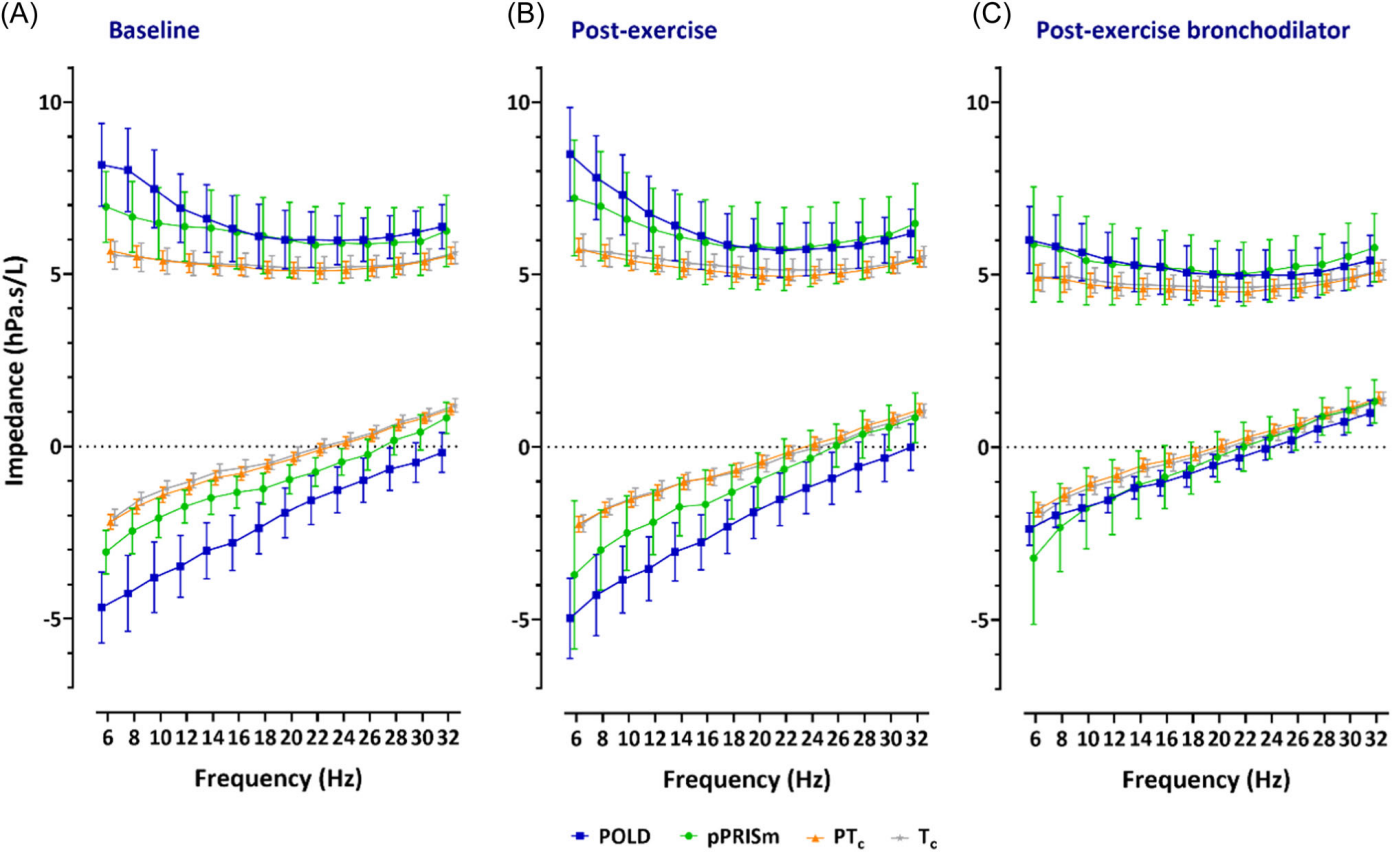
Oscillometry spectra for impedance across frequencies for resistance and reactance (at 6–32 Hz) at baseline, postexercise and after postexercise bronchodilator, for preterm‐associated obstructive lung disease (POLD), preterm‐associated preserved ratio of impaired spirometry (pPRISm), preterm (PT_c_), and term (T_c_) controls.

**Figure 3:**
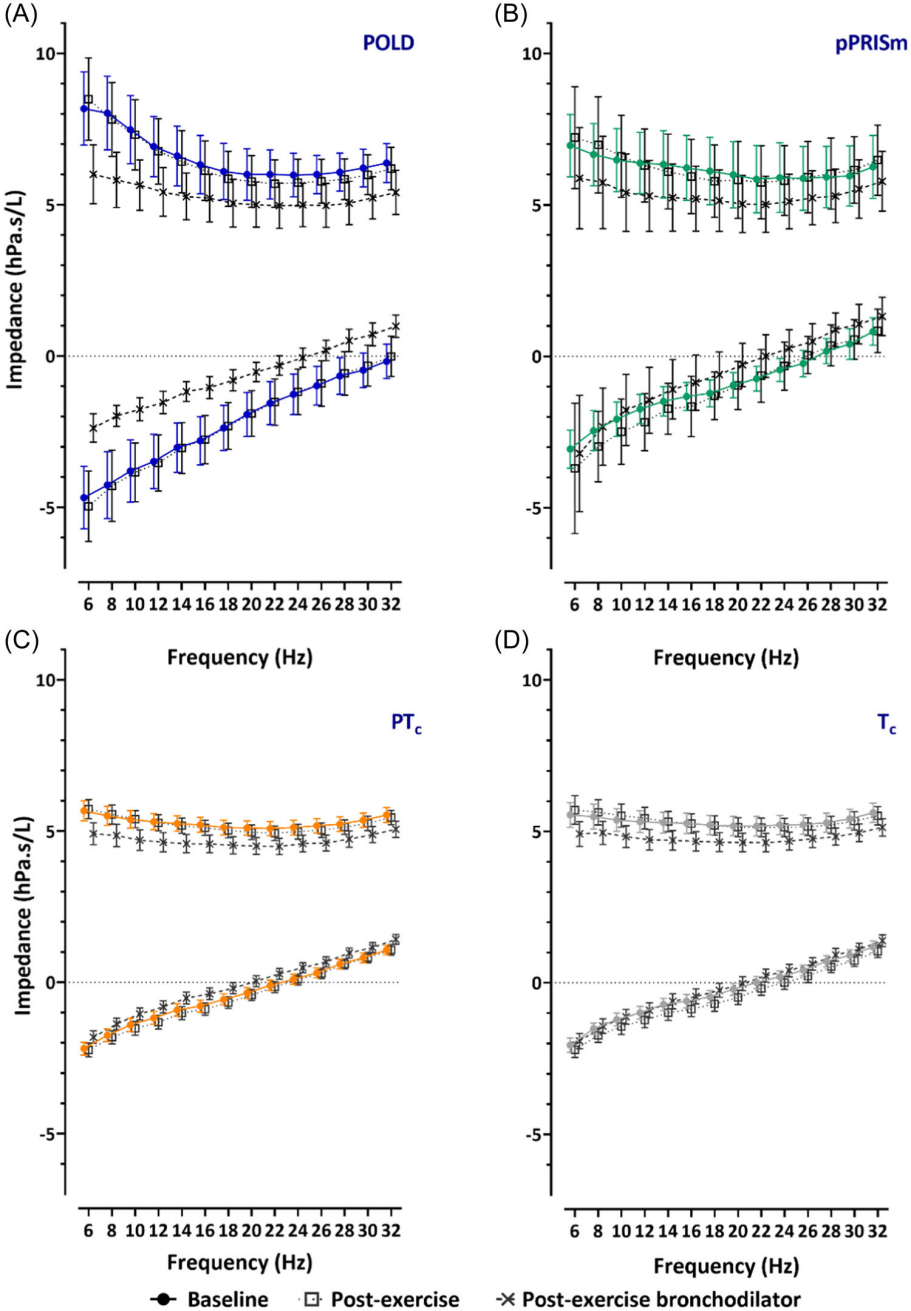
Oscillometry spectra for impedance across frequencies for resistance and reactance (at 6–32 Hz) for prematurity‐associated obstructive lung disease (POLD), prematurity‐associated preserved ratio of impaired spirometry (pPRISm), preterm (PT_c_), and term (T_c_) controls, at baseline, postexercise, and after postexercise bronchodilator.

Oscillometry measurements are shown in Table 2 and graphically in Figures 2 and 3. Differences between baseline and after exercise; and between exercise and postexercise bronchodilator measurements are shown in Tables 3 and 4.

**Table 2:** Oscillometry results for preterm obstructive lung disease (POLD), preterm preserved ratio impaired spirometry (pPRISm), preterm (PT_c_), and term (T_c_) controls.

	POLD (*n* = 15)	pPRISm (*n* = 11)	PT_c (_ *n* = 93)	T_c_ (*n* = 60)
Breathing frequency, breaths per minutes				
Baseline	23.4 (20.2 to 26.6)	20.9 (17.3 to 24.5)	20.2 (19.0 to 21.5)	19.7 (18.1 to 21.3)
Postexercise	23.7 (19.9 to 27.5)	24.5 (20.2 to 28.8)	^∂∂^ 22.4 (20.9 to 23.9)	^∂∂^ 22.3 (20.4 to 24.2)
Postexercise BD	26.5 (22.6 to 30.4)	25.8 (21.4 to 30.2)	23.2 (21.7 to 24.8)	22.2 (20.3 to 24.2)
**Resistance parameters**				
Rrs_mean_, hPa.s/L				
Baseline	**7.0** (**6.0 to 8.0)^††† ‡‡‡^**	6.4 (5.3 to 7.4)	5.3 (5.1 to 5.6)	5.3 (5.0 to 5.7)
Postexercise	**6.8** (**5.8 to 7.9)^†† ‡‡^**	6.3 (5.0 to 7.7)	5.3 (5.0 to 5.5)	5.4 (5.0 to 5.8)
Postexercise BD	^₸₸₸^ 5.4 (4.6 to 6.2)	^₸₸^ 5.4 (4.2 to 6.6)	^₸₸₸^ 4.7 (4.4 to 5.0)	^₸₸₸^ 4.8 (4.4 to 5.1)
Rrs_6_, hPa.s/L				
Baseline	**8.2** (**7.0 to 9.4)^††† ‡‡‡^**	7.0 (5.9 to 8.0)	5.7 (5.3 to 6.0)	5.5 (5.1 to 6.0)
Postexercise	**8.5** (**7.1 to 9.8)^††† ‡‡‡^**	7.2 (5.5 to 8.9)	5.7 (5.4 to 6.0)	5.7 (5.2 to 6.2)
Postexercise BD	^₸₸₸^ 6.0 (5.0 to 7.0)	^₸₸^ 5.9 (4.2 to 7.6)	^₸₸₸^ 4.9 (4.6 to 5.3)	^₸₸₸^ 4.9 (4.5 to 5.3)
Rrs_6_, z‐score				
Baseline	**1.45** (**0.96 to 1.95)^††† ‡‡‡^**	**0.87** (**0.29 to 1.45)^¶¶^**	0.07 (−0.13 to 0.27)	−0.24 (−0.49 to 0.01)
Baseline z‐score >ULN	**6** (**40%)^††† ‡‡‡^**	0 (0%)	1 (1%)	1 (2%)
Postexercise	**1.58** (**1.00 to 2.15)^††† ‡‡‡^**	**0.90** (**0.24 to 1.57)** ^¶^	0.12 (−0.11 to 0.35)	−0.15 (−0.44 to 0.14)
Postexercise BD	0.01 (−0.65 to 0.66)	−0.12 (−0.88 to 0.65)	−0.66 (−0.92 to −0.40)	−0.83 (−1.15 to −0.50)
Rrs_20_, hPa.s/L				
Baseline	6.0 (5.2 to 6.8)	6.0 (4.9 to 7.1)	5.1 (4.9 to 5.3)	5.2 (4.9 to 5.5)
Postexercise	5.8 (4.9 to 6.6)	5.8 (4.6 to 7.1)	5.0 (4.7 to 5.2)	5.1 (4.8 to 5.5)
Postexercise BD	^₸₸₸^ 5.0 (4.3 to 5.8)	^₸₸^ 5.0 (4.1 to 6.0)	^₸₸₸^ 4.5 (4.2 to 4.8)	^₸₸₸^ 4.6 (4.3 to 4.9)
Rrs_6–20_, hPa.s/L				
Baseline	**2.2** (1.5 to 2.9)^** ††† ‡‡‡^	1.0 (0.5 to 1.4)	0.6 (0.4 to 0.8)	0.4 (0.2 to 0.6)
Postexercise	**2.7** (**1.9 to 3.5)** ^* **††† ‡‡‡**^	1.4 (0.5 to 2.3)	0.8 (0.6 to 1.0)	0.6 (0.3 to 0.8)
Postexercise BD	^₸₸₸^ 1.0 (0.5 to 1.5)	0.9 (−0.2 to 1.9)	^₸₸^ 0.4 (0.2 to 0.6)	0.3 (0.0 to 0.5)
**Reactance parameters**				
Xrs_6_, hPa.s/L				
Baseline	−**4.7** (−**5.7 to** −**3.6**)^*** ††† ‡‡‡^	−**3.1** (−**3.7 to** −**2.4)** ^¶^	−2.2 (−2.4 to −2.0)	−2.1 (−2.3 to −1.8)
Postexercise	−**5.0** (−**6.1 to** −**3.8)^††† ‡‡‡^**	−**3.7** (−**5.8 to** −**1.6)^¥¥ ¶¶^**	−2.2 (−2.5 to −2.0)	−2.2 (−2.5 to −2.0)
Postexercise BD	^₸₸₸^−2.4 (−2.8 to −1.9)	−**3.2** (−**5.1 to** −**1.3)^¥¥ ¶¶^**	^₸₸^−1.8 (−2.0 to −1.6)	−1.9 (−2.2 to −1.7)
Xrs_6_, z‐score				
Baseline	**2.55** (2.08 to 3.02)^*** ††† ‡‡‡^	**1.07** (**0.52 to 1.62)^¶¶^**	0.31 (0.12 to 0.5)	0.03 (−0.21 to 0.26)
Baseline z‐score >ULN	**11** (73%)^** ††† ‡‡‡^	1 (9%)	7 (8%)	2 (3%)
Postexercise	**2.81** (**2.21 to 3.41)^††† ‡‡‡^**	**1.58** (**0.87 to 2.28)^¥¥ ¶¶^**	0.35 (0.11 to 0.6)	0.19 (−0.12 to 0.49)
Postexercise BD	0.30 (−0.24 to 0.84)	**1.11** (**0.48 to 1.74)^¥¥ ¶¶^**	−0.09 (−0.31 to 0.12)	−0.12 (−0.39 to 0.15)
Xrs_20_, hPa.s/L				
Baseline	−**1.9** (−**2.7 to** −1.2)* **^††† ‡‡‡^**	−1.0 (−1.4 to −0.5)	−0.3 (−0.5 to −0.2)	−0.2 (−0.4 to 0.0)
Postexercise	−**1.9** (−**2.6 to** −**1.1)^††† ‡‡‡^**	−1.0 (−1.8 to −0.2)	−0.4 (−0.6 to −0.2)	^∂∂^−0.5 (−0.7 to −0.2)
Postexercise BD	^₸₸₸^−0.5 (−0.9 to −0.2)	^₸₸^−0.3 (−1.0 to 0.4)	^₸₸₸^ 0.0 (−0.1 to 0.2)	^₸₸₸^ 0.0 (−0.3 to 0.2)
Crs, mL/hPa				
Baseline	**5.4** (**4.1 to 6.8)^††† ‡‡‡^**	**7.9** (**6.3 to 9.5)** ^¶^	11.3 (10.3 to 12.2)	11.9 (10.7 to 13.0)
Postexercise	**5.2** (**4.0 to 6.4)^††† ‡‡‡^**	7.6 (5.3 to 10.0)	11.0 (10.1 to 11.9)	11.2 (9.9 to 12.5)
Postexercise BD	^₸₸₸^ 9.4 (7.8 to 11.0)	₸10.3 (6.9 to 13.7)	^₸₸₸^ 13.5 (12.3 to 14.6)	^₸₸₸^ 13.1 (11.5 to 14.6)
*ƒ*res, Hz				
Baseline	**33.8** (**28.9 to 38.7)^††† ‡‡‡^**	26.6 (23.5 to 29.7)	22.5 (21.1 to 23.9)	21.7 (20.0 to 23.4)
Postexercise	**31.1** (**27.2 to 35.1)^††† ‡‡^**	25.0 (21.4 to 28.6)	23.0 (21.5 to 24.6)	23.3 (21.5 to 25.1)
Postexercise BD	^₸₸₸^ **24.6** (**21.6 to 27.6)** ^†^	^₸₸^ 20.6 (16.8 to 24.4)	^₸₸₸^ 19.4 (18 to 20.9)	^₸₸₸^ 20.0 (18.2 to 21.9)
AX, hPa/L				
Baseline	**62.6** (43.5 to 81.8)^*** ††† ‡‡‡^	29.6 (19.3 to 39.9)	19.1 (15.4 to 22.9)	16.1 (12.5 to 19.7)
Postexercise	**61.4** (**40.4 to 82.4)** ^* **††† ‡‡‡**^	35.2 (14.9 to 55.5)	20.9 (17.0 to 24.9)	20.4 (16.1 to 24.7)
Postexercise BD	^₸₸₸^ 22.1 (15.3 to 28.9)	₸23.1 (4.8 to 41.4)	^₸₸₸^ 13.7 (10.5 to 16.9)	₸14.7 (10.9 to 18.5)
AX, z‐score				
Baseline	**2.46** (1.93 to 3.00)^** ††† ‡‡‡^	**0.87** (**0.24 to 1.49)^¶¶^**	0.12 (−0.09 to 0.34)	−0.24 (−0.51 to 0.02)
Baseline z‐score >ULN	**9** (**60%)^††† ‡‡‡^**	1 (9%)	4 (4%)	1 (2%)
Postexercise	**2.36** (**1.73 to 2.98)** ^* **††† ‡‡‡**^	1.03 (0.30 to 1.76)	0.25 (0.00 to 0.51)	0.07 (−0.24 to 0.38)
Postexercise BD	0.30 (−0.28 to 0.87)	0.12 (−0.55 to 0.80)	−0.39 (−0.62 to −0.16)	−0.43 (−0.72 to −0.15)

*Note*: Results expressed as mean and 95% confidence intervals for continuous data (two‐way ANOVA with Bonferroni correction).

Significance symbols: *POLD vs. pPRISm, ^†^POLD vs. PTc, ^‡^POLD vs. Tc, ^¥^pPRISm vs. PTc, ^¶^PRISm vs. Tc, ^₴^PTc vs. Tc; ^∂^Baseline vs. postexercise, ^₸^Postexercise vs. postbronchodilator (Single symbol denotes significance level <0.05, double symbol <0.01, triple symbol <0.001).

(“Single symbol” denotes significance level <0.05, “double symbol” <0.01, “triple symbol” <0.001. Bold values indicate statistical significance between groups. Underlined values indicate statistical significance between time points.).

Abbreviations: ANOVA, analysis of variance; AX, area above reactance curve between 6 Hz and fres; Crs, compliance; *ƒ*res, resonant frequency; Rrs_mean_, average respiratory system resistance 6–20 Hz; R/Xrs_6/20_, respiratory system resistance (Rrs)/reactance (Xrs) at 6/20 Hz; Rrs_6–20_, frequency dependence of resistance between 6 and 20 Hz; ULN,upper limit of normal.

**Table 3:** Oscillometry change scores from baseline to postexercise for preterm obstructive lung disease (POLD), preterm preserved ratio impaired spirometry (pPRISm), preterm (PTc), and term (Tc) controls.

	POLD (*n* = 15)	pPRISm (*n* = 11)	PT_c_ (*n* = 93)	T_c_ (*n* = 60)
**Resistance parameters**				
Rrs_mean_				
Absolute (hPa.s/L)	−0.14 (−0.54 to 0.27)	−0.05 (−0.72 to 0.62)	−0.04 (−0.22 to 0.13)	0.07 (−0.19 to 0.32)
Relative (%)	−1.85 (−9.18 to 5.47)	−1.6 (−12.06 to 8.85)	0.26 (−2.97 to 3.49)	2.7 (−2 to 7.4)
Rrs_6_				
Absolute (hPa.s/L)	0.31 (−0.43 to 1.06)	0.26 (−0.72 to 1.25)	0.06 (−0.19 to 0.3)	0.16 (−0.16 to 0.48)
Relative (%)	4.29 (−5.87 to 14.45)	2.3 (−10.27 to 14.87)	3.04 (−1.29 to 7.37)	4.39 (−1.68 to 10.46)
Rrs_20_				
Absolute (hPa.s/L)	−0.23 (−0.56 to 0.09)	−0.17 (−0.79 to 0.45)	−0.14 (−0.3 to 0.02)	−0.04 (−0.28 to 0.2)
Relative (%)	−3.81 (−10.26 to 2.65)	−3.63 (−15.8 to 8.54)	−2.06 (−5.06 to 0.94)	0.64 (−3.83 to 5.11)
Rrs_6−20_, hPa.s/L (absolute)	0.55 (−0.05 to 1.14)	0.43 (−0.28 to 1.15)	0.20 (0.02 to 0.37)	0.20 (−0.06 to 0.47)
**Reactance parameters**				
Xrs_6_, hPa.s/L (absolute)	0.29 (−0.45 to 1.03)	0.64 (−1.14 to 2.41)	0.05 (−0.14 to 0.23)	0.16 (−0.05 to 0.36)
Xrs_20_, hPa.s/L (absolute)	−0.03 (−0.39 to 0.33)	0.01 (−0.46 to 0.48)	0.07 (−0.07 to 0.2)	0.27 (0.08 to 0.46)
Crs				
Absolute (mL/hPa)	−0.23 (−1.08 to 0.62)	−0.27 (−1.77 to 1.24)	−0.31 (−0.95 to 0.34)	−0.67 (−1.61 to 0.28)
Relative (%)	0.29 (−16.03 to 16.62)	−5.36 (−27.03 to 16.31)	0.29 (−4.9 to 5.48)	−3.25 (−10.42 to 3.91)
*f*res				
Absolute, Hz	−2.62 (−5.23 to −0.02)	−1.6 (−3.64 to 0.43)	0.55 (−0.63 to 1.73)	1.58 (−0.05 to 3.2)
Relative (%)	−6.68 (−13.13 to −0.23)	−6.09 (−14.01 to 1.82)	5.18 (−0.49 to 10.85)	10.83 (2.33 to 19.34)
AX				
Absolute (hPa/L)	−1.24 (−10.33 to 7.86)	5.63 (−5.88 to 17.14)	1.80 (−0.85 to 4.45)	4.32 (0.73 to 7.92)
Relative (%)	−1.7 (−19.53 to 16.13)	6.9 (−20.57 to 34.38)	38.36 (9.04 to 67.68)	69.76 (21.53 to 118)

*Note*: Results expressed as mean and 95% confidence intervals for continuous data (one‐way ANOVA with Bonferroni correction).

Significance symbols: *POLD vs. pPRISm, ^†^POLD vs. PTc, ^‡^POLD vs. Tc, ^¥^pPRISm vs. PTc, ^¶^PRISm vs. Tc, ^₴^PTc vs Tc.

(“Single symbol” denotes significance level <0.05, “double symbol” <0.01, “triple symbol” <0.001.)

Abbreviations: ANOVA, analysis of variance; AX, area above reactance curve between 6 Hz and fres; Crs, compliance; *ƒ*res, resonant frequency; Rrs_mean_, average respiratory system resistance 6–20 Hz; R/Xrs_6/20_, respiratory system resistance (Rrs)/reactance (Xrs) at 6/20 Hz; Rrs_6–20_, frequency dependence of resistance between 6 and 20 Hz.

### 3.4 Resistance

At baseline (Table 2), Rrs_mean_ was 1.3‐fold greater in magnitude in the POLD group compared to the preterm and term control groups (7.0 vs. 5.3 vs. 5.3 hPa.s/L) and Rrs_6_ was 1.4‐ and 1.5‐fold greater in magnitude in the POLD group compared to the preterm and term controls, respectively (8.2 vs. 5.7 vs. 5.5 hPa.s/L). There was a significantly greater Rrs_6–20_ seen in POLD compared to pPRISM and both control groups of 2.2‐, 3.7‐, and 5.5‐fold greater magnitudes, respectively (2.2 vs. 1.0 vs. 0.6 vs. 0.4 hPa.s/L). Rrs_6_ z‐score showed similar differences to the raw values, with children with POLD having significantly greater scores than preterm and term controls (1.45 vs. 0.07 vs. −0.24). Additionally, 40% of the POLD group had a baseline z‐score greater than the upper limit of normal, compared to 0%, 1%, and 2% of the pPRISm, and in the preterm and term groups.

Following exercise, there were no statistically significant changes noted in oscillometry variables (Table 3). There were no differences in either relative or absolute magnitude of change between the groups.

Following postexercise bronchodilator administration, all groups had significant improvements in almost all resistance parameters (Table 4), with the exception of Fdep in the pPRISm and T_c_ groups. The greatest improvements were in children with POLD, with significantly greater differences observed for absolute Rrs_mean_, Rrs_6_, and Rrs_6–20_ compared to both control groups. The decrease in absolute Rrs_6_ following bronchodilator was approximately 300% greater for the POLD when compared to the preterm and term groups. 20% of the POLD group had a positive bronchodilator response in Rrs_6_ with fewer (9% vs. 8% vs. 5%) noted in the in pPRISm, PT_c_ and T_c_ groups, respectively (Table 4).

**Table 4:** Oscillometry change scores from postexercise to postexercise bronchodilator for preterm obstructive lung disease (POLD), preterm preserved ratio impaired spirometry (pPRISm), preterm (PTc), and term (Tc) controls.

	POLD (*n* = 15)	pPRISm (*n* = 11)	PT_c_ (*n* = 93)	T_c_ (*n* = 60)
**Resistance parameters**				
Rrs_mean_				
Absolute (hPa.s/L)	**−1.39** (**−2.07 to −0.71)** ^†‡^	**−**0.98 (**−**1.49 to **−**0.46)	**−**0.62 (**−**0.8 to **−**0.44)	**−**0.64 (**−**0.84 to **−**0.45)
Relative (%)	**−**18.28 (**−**28.5 to **−**8.07)	**−**15.11 (**−**21.72 to **−**8.49)	**−**12.04 (**−**15.14 to **−**8.94)	**−**11.33 (**−**14.82 to **−**7.83)
Rrs_6_				
Absolute (hPa.s/L)	**−2.48** (**−3.54 to −1.43)^††† ‡‡‡^**	**−**1.34 (**−**2.55 to **−**0.13)	**−**0.82 (**−**1.07 to **−**0.56)	−0.79 (**−**1.11 to **−**0.47)
Change >40% (%)	3 (20%)	1 (9%)	7 (8%)	3 (5%)
Relative (%)	**−**26.53 (**−**38.38 to **−**14.68)	**−**18.45 (**−**31.38 to **−**5.52)	**−**13.99 (**−**17.88 to **−**10.1)	**−**12.01 (**−**16.98 to **−**7.04)
Rrs_20_				
Absolute (hPa.s/L)	**−**0.76 (**−**1.3 to **−**0.22)	−0.79 (**−**1.4 to **−**0.18)	**−**0.45 (**−**0.6 to **−**0.3)	**−**0.51 (**−**0.69 to **−**0.33)
Relative (%)	**−**11.28 (**−**21.64 to **−**0.91)	**−**11.04 (**−**20.38 to **−**1.7)	**−**9.32 (**−**12.17 to **−**6.47)	**−**9.35 (**−**12.76 to **−**5.94)
Rrs_6–20_, hPa.s/L (absolute)	**−1.72** (**−2.53 to −0.91)** ^* **††† ‡‡‡**^	**−**0.55 (**−**1.58 to 0.48)	**−**0.36 (**−**0.54 to **−**0.18)	**−**0.29 (**−**0.53 to **−**0.05)
**Reactance parameters**				
Xrs_6_				
Absolute (hPa.s/L)	**2.59** (1.51 to 3.67)^*** ††† ‡‡‡^	0.49 (**−**0.54 to 1.53)	0.43 (0.22 to 0.64)	0.29 (0.08 to 0.5)
Change >−50% (%)	6 (40%)	1 (9%)	14 (15%)	7 (12%)
Xrs_20_, hPa.s/L (absolute)	**1.37** (**0.68 to 2.05)^††† ‡‡‡^**	0.68 (0.27 to 1.09)	0.45 (0.31 to 0.6)	0.45 (0.32 to 0.59)
Crs				
Absolute (mL/hPa)	4.18 (2.68 to 5.69)	2.7 (0.69 to 4.7)	2.5 (1.74 to 3.26)	1.87 (0.98 to 2.77)
Relative (%)	**96.18** (58.92 to 133.43)^** ††† ‡‡‡^	37.4 (8.18 to 66.63)	27.99 (20.17 to 35.81)	18.98 (11.52 to 26.44)
*f*res				
Absolute, Hz	**−**6.55 (**−**9.85 to **−**3.26)	**−**4.38 (**−**6.2 to **−**2.56)	**−**3.62 (**−**4.63 to **−**2.62)	**−**3.25 (**−**4.46 to **−**2.04)
Relative (%)	**−**19.19 (**−**28.28 to **−**10.1)	**−**17.89 (**−**24.79 to **−**10.99)	**−**15.02 (**−**18.84 to **−**11.19)	**−**12.77 (**−**18.24 to **−**7.31)
AX				
Absolute (hPa/L)	**−39.3** (−57.79 to −20.82)^*** ††† ‡‡‡^	**−**12.11 (**−**21.32 to **−**2.9)	**−**7.42 (**−**10.68 to **−**4.17)	**−**5.68 (**−**8.03 to **−**3.33)
Change >80% (%)	2 (13%)	0 (0%)	7 (8%)	12 (7%)
Relative (%)	**−**55.15 (**−**70.27 to **−**40.03)	**−**41.23 (**−**60.22 to **−**22.23)	**−**29.45 (**−**39.59 to **−**19.31)	**−**21.21 (**−**35.64 to **−**6.78)

*Note*: Results expressed as mean and 95% confidence intervals for continuous data (one‐way ANOVA with Bonferroni correction).

Significance symbols: *POLD vs. pPRISm, ^†^POLD vs. PTc, ^‡^POLD vs. Tc, ^¥^pPRISm vs. PTc, ^¶^PRISm vs. Tc, ^₴^PTc vs. Tc.

(“Single symbol” denotes significance level <0.05, “double symbol” <0.01, “triple symbol” <0.001. Bold values indicate statistical significance between groups.).

Abbreviations: ANOVA, analysis of variance; AX, area above reactance curve between 6 Hz and fres; Crs, compliance; *ƒ*res, resonant frequency; Rrs_mean_, average respiratory system resistance 6–20 Hz; R/Xrs_6/20_, respiratory system resistance (Rrs)/reactance (Xrs) at 6/20 Hz; Rrs_6–20_, frequency dependence of resistance between 6 and 20 Hz.

### 3.5 Reactance

Reactance values were significantly worse (more negative) in the POLD group at baseline compared to the children with pPRISm and both control groups (Xrs_6_: −4.7 vs. −3.1 vs. −2.2 vs. −2.1 hPa.s/L; Xrs_20_: −1.9 vs. −1.0 vs. −0.3 vs. −0.2), with more negative Xrs_6_ also seen in the pPRISm group when compared to term controls. This was reflected in low Crs values for the POLD (compared to both controls 5.4 vs. 11.3 vs. 11.9 mL/hPa) and the pPRISm groups (7.9 mL/hPa vs. term controls). *ƒ*res was significantly higher in the POLD group when compared to the preterm and term groups (33.8 vs. 22.5 vs. 21.7 Hz). AX in children with POLD (62.6 hPa/L) was approximately twofold greater compared to the pPRISm group (29.6), threefold greater than the preterm group (19.1 hPa/L) and almost fourfold greater than in the term group (16.1 hPa/L).

Following exercise, the only statistically significant change observed was a small change in Xrs_20_ in the term group.

Following postexercise bronchodilator administration, improvements were noted for all oscillometry parameters in all four groups, with the exception of Xrs_6_ in the pPRISm group. The greatest changes were seen in the POLD group, as reflected in their absolute change in scores after postexercise bronchodilator for Xrs_6_, AX, and relative change score for Crs when compared to the pPRISm, PT_c_ and T_c_ groups. A total of 40% and 13% of the POLD group had positive bronchodilator response for Xrs_6_ and AX, respectively, with fewer (9%, 15%, and 15% for Xrs_6_; and 0%, 8%, and 7% for AX) noted in the in pPRISm, PT_c_ and T_c_ groups, respectively (Table 4).

#### 3.5.1 Sensitivity analyses

Since we had included preterm controls with FEV_1_ > LLN but term controls had percent predicted FEV_1_ > 90%, we investigated if these differences impacted on the results. We compared the preterm control group, but only those with percent predicted FEV_1_ > 90% (in line with term controls), with the original PT_c_ group. As shown in Table SE2 (online Supporting Information), the original and revised PT_c_ groups appear to have similar oscillometry data, with a maximum difference of 0.2 hPa.s/L in any of the impedance measurements between the preterm control group as a whole and just those with %FEV_1_ > 90%, and thus any differences compared to POLD and pPRISm remain.

## 4 DISCUSSION

The present study has assessed the mechanical properties of the respiratory system using oscillometry, including responses to exercise and postexercise bronchodilator administration in preterm‐ and term‐born children. We have demonstrated that preterm‐born children with obstructive lung disease (POLD group) had impaired baseline respiratory mechanics when compared to preterm‐born children with preserved ratio of impaired spirometry (pPRISm group), and preterm‐ and term‐born controls with normal spirometry. We noted that exercise did not have a major effect on oscillometry variables in either preterm‐ or term‐born children. However, both preterm‐ and term‐born children showed varying degrees of postexercise bronchodilator response. The greatest responses were observed in preterm‐born children with obstructive airway disease.

Spirometry has been shown to correlate with oscillometry,^31^, ^32^ thus our identification of impaired respiratory mechanics in a group of preterm‐born children stratified by lung function is perhaps unsurprising. However, as spirometry cannot be performed reliably in younger children, oscillometry has the potential to identify preterm‐born children who have impaired lung function at a much earlier stage allowing satisfactory treatment to be instituted. Additionally, oscillometry has been successfully used to differentiate obstructive lung disease in cystic fibrosis and asthma in children^13^ thus certainly has the potential to identify obstructive lung disease in preterm‐born children and potentially infants.

Baseline oscillometry revealed that those in the POLD group were most affected, with intermediate values noted in the pPRISm group, whilst the preterm control group showed few differences to term‐born children. Oscillometry in other preterm populations have shown, as a group, preterm‐born children have impaired impedance^33^; this potentially may be driven by those preterm‐born children with an obstructive or pPRISm phenotype of lung dysfunction (rather than specifically CLD), with other preterm‐born children within that population having impedance towards normality. This outlines the importance not to consider preterm‐born children as a single‐entity, but to classify then into different phenotypes. The largest differences were observed at the lower frequencies suggesting that peripheral lung disease is the major site of airway affliction in these children. This is consistent with findings in other obstructive lung diseases such as in uncontrolled asthma.^34^ Reactance, particularly at the lower frequencies, i.e., elastic properties of the respiratory system, also appeared to be impaired; indeed, this was reflected in low Crs values, especially for the POLD group (approximately half that of either preterm or term children with normal lung function). Poor lung compliance is a major factor in neonatal lung disease, including in neonatal respiratory distress syndrome and in early infancy with the diagnosis of CLD,^35^ with the hypothesis proposed that hyperinflation leads to stiffer lungs as reflected by impaired compliance.^36^ Our findings suggest that compliance remains impaired in a subgroup of children and appears not to be linked to the diagnosis of CLD in infancy, since a significant proportion of the children in our cohort with impaired spirometry and oscillometry measures did not have CLD in infancy. Another consideration for the low Crs values in children with POLD could be the diseased areas of the lungs causing an uneven time constant distribution, and a resulting frequency dependence of compliance.

There is an absence of published data on airway mechanics in (p)PRISm. Our population showed a degree of impaired impedance, less than the obstructive population, and without the same peripheral airway involvement as seen in the children with POLD. Additionally, they had limited bronchodilator responsiveness suggesting fixed airway disease. This population of children are less likely to have modifiable disease process; however, close surveillance is likely to be necessary given the longer‐term outcomes associated with increased mortality and morbidity in adulthood.

Bronchodilator use has previously been assessed in a range of respiratory conditions using oscillometry including in preterm‐born children.^14^ We noted the greatest response to postexercise bronchodilator in the obstructive group, in particular, for reactance, suggesting that bronchodilators improve compliance, potentially as a result of reduced ventilation inhomogeneity. Given the greater decrease in resistance observed at lower frequencies in the obstructive group, as demonstrated by the frequency‐dependence of resistance, this suggests that bronchodilators are most likely acting on the peripheral airways. A mechanistic possibility is of smooth muscle extending distally as a result of repair and remodeling after preterm birth, which suggests reversible disease that may be amenable to appropriate treatment with beta_2_ agonists.^37^ This potential, along with the hypothesis of lungs secondary to air‐trapping, is a conceivable explanation for the underlying disease mechanism. These observations reinforce the need to identify preterm‐born children with decreased lung function into different phenotypes that respond differently to inhaled medication. It is likely, given the lack of exercise‐induced decrements in oscillometry findings, that improvements seen with oscillometry following postexercise bronchodilator are due to improvement in baseline bronchoconstrictor tone, which could be confirmed by oscillometry before and after administration of bronchodilator at baseline. Fewer children had positive bronchodilator response using oscillometry criteria (40%, 50%, and 80% for Rrs_6_, Xrs_6_, and AX, respectively) compared to 94% of children with POLD who increased their %FEV_1_ > 10%^20^ suggesting that further assessment is required before these criteria can be used in clinical practice, especially in younger children who are unable to do spirometry.

Also of interest were the oscillometry measures following exercise. A greater number of children from the POLD group did not achieve maximal exercise testing, and those children with POLD had lower exercise capacity,^20^ which may be a mixture of impaired lung function, respiratory symptoms and deconditioning.^38^ While exercise is known to potentially cause bronchoconstriction confirmed by spirometry in preterm children,^39^ its impact on respiratory mechanics has not been reported previously. We have shown that exercise does not appear to have a major effect on respiratory mechanics in either preterm‐ or term‐born children, including those with obstructive airway disease, with only small, nonstatistically significant changes noted. In terms of exercise‐induced bronchoconstriction, at present there are no defined cut‐offs for oscillometry to allow an estimation of the number of individuals within each group who had evidence of exercise‐induced bronchoconstriction; only two of these children showed evidence for postexercise bronchoconstriction with spirometry testing.^20^ Oscillometry cut‐offs to determine thresholds for postexercise bronchoconstriction would be welcome. Potentially serial postexercise oscillometry may show that a maximal change is at a different time point to that observed for spirometry.

There are several possible explanations why little change was noted after exercise: oscillometry was performed at 20 min after exercise when exercise‐induced bronchoconstriction (EIB), as measured by spirometry, is generally at its peak,^39^ although spirometry and oscillometry findings regarding timing of exercise‐induced bronchoconstriction may not be interchangeable. It is possible that any effects of exercise on the lung mechanics were present at an earlier time after the exercise test completed; however, the corresponding spirometry results also did not show any EIB. It may be that oscillometry did not record any changes after exercise due to the timing of the measurements; earlier measurements after 5–10 min of stopping exercise may be more appropriate as recommended by the recent ERS standards.^40^ Alternatively, using either a treadmill or single bouts of submaximal exercise may induce greater bronchoconstriction.

### 4.1 Strengths and limitations

The main strength of this study is assessing functional outcomes based on a current measure of lung function rather than a historical diagnosis of CLD. Additionally, while it is common in other lung diseases to classify the disease process using spirometry into obstructive or restrictive disease processes, this has been utilized infrequently in preterm‐born children with respiratory compromise. Furthermore, we have described features in children with pPRISm, a clinical phenotype which is associated with significant morbidity and mortality in adulthood.^17^, ^18^ One limitation regarding oscillometry is use of predominantly raw values rather than z‐scores derived from satisfactory reference ranges; however, the variables for which we were able to use z‐scores showed results which were consistent with the raw values. Additionally, we included a sufficiently large term control group to counter the lack of robust population‐based references values against which our data could have been standardized. Despite the small numbers available in the POLD and pPRISm groups, we were able to show important differences between the groups of interest. We acknowledge that larger, possibly collaborative studies, are required for these observations to be made more generalizable. Additionally, the cut‐off values for bronchodilator response as used above are from small number of reference children, and more robust, possibly population specific, cut‐offs for response would be of greater use, but currently are not available. A further area of interest would be exploring whether postexercise changes are detectable with oscillometry at an alternative time point following maximal exercise testing.

## 5 CONCLUSIONS

In summary, preterm‐born children with low lung function who have obstructive airway disease have greater impairment of their respiratory mechanics compared to preterm‐ and term‐born children with normal spirometry. The oscillometry results show that peripheral airway disease appears to be present in children with POLD, and that their airways were responsive to postexercise bronchodilator administration, resulting in significantly improved lung compliance. Although the exact mechanism underlying lung dysfunction (structural vs. inflammation) still needs to be clarified, we have shown that bronchodilators do appear to improve the airflow limitation noted in children with POLD. With the findings of combined long‐acting beta‐2 agonists with inhaled corticosteroids improving spirometry parameters in preterm‐born children,^22^ consideration of treatment in these children is required.

## AUTHOR CONTRIBUTIONS

**Michael Cousins**: data curation; formal analysis; writing—original draft; writing—review & editing; investigation. **Kylie Hart**: data curation; investigation; writing—review & editing. **Bence L Radics**: writing—review & editing; software. **A John Henderson**: conceptualization. **Zoltán Hantos**: software; resources; writing—review & editing; writing—original draft; supervision. **Sailesh Kotecha**: writing—review & editing; writing—original draft; conceptualization; methodology; resources; funding acquisition; supervision.

## CONFLICT OF INTEREST STATEMENT

SK reports securing a research grant from the Medical Research Council for this work. SK reports funding from HTA/NIHR, Moulton Foundation, GSK, Nutricia Foundation and Aspire Pharma outside this work. BLR and ZH were supported by the Hungarian Scientific Research Fund (Grants K105403 and K128701). BLR, PDS and ZH were supported by the Clinical Research Collaboration award (The International Collaboration to Improve Respiratory Health in Children (INCIRCLE)) by the European Respiratory Society (Grant ERS CRC‐2013‐02). The other authors declare no conflicts of interest.

## Supporting information

Supporting information.

## Data Availability

The data that support the findings of this study are available on request from the corresponding author. The data are not publicly available due to privacy or ethical restrictions.
