# Supplementary material for: Peripheral airway dysfunction in prematurity‐associated obstructive lung disease identified by oscillometry
Source: Pediatr Pulmonol. 2023 Sep 13;58(11):3279–92. doi: 10.1002/ppul.26658 (PMC10947003; doi:10.1002/ppul.26658)
Supplement: Supplementary file 1 — Supporting information. [file PPUL-58-3279-s001.DOC]

Peripheral airway dysfunction in prematurity-associated obstructive lung disease identified by oscillometry

^1,2^Michael Cousins MRCPCH, PhD, ^1,2^Kylie Hart MSc, ^3^Bence Radics MD, ^4^A John Henderson FRCPCH, PhD, ^5^Zoltán Hantos PhD DSc, ^6^Peter D Sly MD DSc, ^2^Sailesh Kotecha FRCPCH, PhD.

^1^Department of Child Health, Cardiff University School of Medicine, Cardiff, United Kingdom.

^2^Department of Paediatrics, Cardiff and Vale University Health Board, Cardiff, United Kingdom.

^3^Department of Pathology, University of Szeged, Szeged, Hungary.

^4^MRC Integrative Epidemiology Unit, Population Health Sciences, Bristol Medical School, University of Bristol, Bristol, United Kingdom.

^5^Department of Anesthesiology and Intensive Therapy, Semmelweis University, Budapest, Hungary.

^6^Child Health Research Centre, The University of Queensland, South Brisbane, Australia.

**This publication is dedicated to our very dear, late friend Professor John Henderson.

**Corresponding Author:** Professor Sailesh Kotecha

Department of Child Health

Cardiff University School of Medicine

Cardiff CF14 4XN

United Kingdom

Email: [KotechaS@cardiff.ac.uk](mailto:KotechaS@cardiff.ac.uk)

# Methods

## **Recruitment of participants**

Children aged 7-12 years were recruited as part of the wider Respiratory Health outcomes in NeOnates (RHiNO) study (EudraCT: 2015-003712-20). Children born preterm between 2003 and 2011, along with term controls, identified during a previous questionnaire study^1^, were contacted regarding participation. Preterm-born children born at ≤34 weeks’ gestation, aged 7-12 years, and geographically accessible were eligible. Children with congenital or cardiorespiratory abnormalities, or significant neurodevelopmental impairment were excluded. The children were sent an initial questionnaire inviting them to participate in RHiNO as previously described ^2,3^. Responders were invited for a screening assessment including spirometry (Microloop Spirometer, Vyaire, Germany) pre- and post-bronchodilator. RHiNO included a randomised control trial of inhalers ^2^, with all those born preterm with %FEV_1_ ≤85% invited for potential RCT recruitment, and the first ten assessments each month of preterm-born children with %FEV_1_>85% together with all term-born children with %FEV_1_ ≥90% as controls, were invited for in-depth study at the Children’s Hospital in Cardiff, UK. Assessment included oscillometry, spirometry, maximal exercise testing, and static lung volumes over 6-7 hours as described.

**Oscillometry testing**

Oscillometry testing was performed using a custom-built set-up and computer programme (NDAQ) developed by a team at University of Szeged in Hungary (Figure E1). The set-up comprised of a loudspeaker connected to a measurement head with sensors to measure pressure and flow at the airway opening. Due to the potential of increased breathing frequency and pressure from exercise the loudspeaker was encased within a larger, sealed cylinder with a shunt tube connecting the top of the speaker to the cylinder below the speaker, allowing pressure to equalise above and below the speaker diaphragm at breathing frequencies.

The test was performed with the child sitting upright in a chair and attached to the system via a Microgard II microbial filter (Vyaire, Germany). A nose clip was worn during testing and children were instructed to firmly hold their cheeks with their fingers and palms of hands to stop any soft tissue vibrations affecting the results. If unable to comply a parent or research team members performed this. A loudspeaker then generated a pseudorandom signal containing even frequencies between 4 and 32 Hz. Impedance was measured at the mouth using the pressure and flow sensors, for each of the individual frequencies. This impedance was measured between the pressure ‘in-phase’ with the input flow signal (representing mostly airway resistance) and the pressure ‘out-of-phase’ with the input signal (representing respiratory reactance). Results from the individual frequencies were displayed in the form of a spectrum. A minimum of 3 recordings were obtained from each child, aiming for spectra that appeared very similar, representing an accurate reflection of respiratory impedance.

Oscillometry was performed at 3 time points. An initial baseline reading was obtained. A second test was performed at 20 minutes following maximal exercise testing. After completing post-exercise spirometry and oscillometry (total 40 minutes after exercise), 400 micrograms of salbutamol (Salamol, TEVA UK Limited) was given via MDI using a Volumatic spacer (GSK, UK). The salbutamol inhaler was shaken before each actuation. Children were instructed to take 10 breaths in and out after each actuation of salbutamol, ensuring the spacer’s valve clicked with each breath. A final oscillometry was performed post-salbutamol.

Post-acquisition analysis was performed of the raw oscillometry data to obtain the results. Each of the recordings was assessed for appropriate quality and artefact-free segments from the 3 recordings (of at least 16 seconds) were selected, compiling results from these using the NDAQ software. The average resistance and reactance results at each individual frequency across the 3 recordings were calculated, plus an average of the resistance measured from all the frequencies between 6 and 20 Hz. Compliance was calculated by fitting an inertance (L) – compliance (C) model to the Xrs data in the 6-32-Hz frequency range (Zrs = R + jωI + (1/jωC), where j is the imaginary unit and ω is angular frequency) ^4^. C and L were determined by the lower-frequency and higher-frequency ranges of reactance, respectively; L is considered a compensatory parameter of less physiological significance and its values are not reported.

## **Spirometry and cardiopulmonary exercise testing (CPET)**

Spirometry was performed using the MasterScreen Body and PFT systems with SentrySuite measurement software version 2.17 (Vyaire Medical, Germany). ERS/ATS guidelines for obtaining suitable spirometry were used for as a guide for performing the test and test acceptability ^5^. An explanation and a demonstration on how to perform the test were done before the child attempted the spirometry. Spirometry was performed with the child sat upright and wearing a nose-clip. They were instructed to take the biggest breath in possible, before blowing out as hard and as fast as they could. Children were vocally encouraged to continue breathing out until they appeared to have reached their residual volume. A minimum of 3 tests were performed, aiming for the intra-test criteria as per Miller *et al*. Spirometry was stopped once satisfactory testing was obtained, if the child did not wish to continue or if the child was unable to perform adequate spirometry. QC was performed to ensure the correct results from all the measurements were used. Daily volume calibrations and weekly flow calibrations were performed using a three-litre syringe. Results were measured at BTSP and Global Lung Initiative predicted values were used to adjust for height, ethnicity, gender and age ^6^. Spirometry was repeated at 4 separated times following conclusion of the exercise test: at 5-10 minutes; 15-20 minutes; 25-30 minutes; 40-45 minutes. This was performed as outlined above. After the final post-exercise spirometry, 400 micrograms of salbutamol (Salamol, TEVA UK Limited) was given via MDI using a Volumatic spacer (GSK, UK). The salbutamol inhaler was shaken before each actuation. Children were instructed to take 10 breaths in and out after each actuation of salbutamol, ensuring the spacer’s valve clicked with each breath. Repeat spirometry was performed 15 minutes after administration of the salbutamol, as described as above.

Cardiopulmonary exercise testing was performed on a Pediatric Cycle Ergometer (Lode, Netherlands) linked to a Masterscreen CPX system (Vyaire Medical, Germany). Children wore a fitted facemask and respiratory parameters were measured using a turbine and gas sampling tube. Data were recorded in a breath-by-breath exercise programme on JLab version 5.72 (Vyaire Medical, Germany). Heart rate was recorded using a Polar H10 heart rate sensor (Polar, UK). Oxygen saturations were monitored with a Nellcor oxygen saturation monitor (Medtronic, USA). A ramp protocol was devised to facilitate the exercise testing. This involved 1 minute of baseline measurements at rest, 3 minutes of minimally-loaded cycling (7 Watts), then at an increasing rate of 1 Watt every 6 seconds (10 Watts per minute). The child was vocally encouraged to continue exercise until they could no longer consistently maintain cadence >60 rpm, with increasing encouragement as the load got higher. Perceived exertion rating was obtained every 3 minutes and at the point the child could no longer continue. 2 minutes of minimally loaded pedalling concluded the test. A test was deemed to be ‘maximal’ if it met ≥2/4 of the following criteria: RER >1.00; HR ≥80% predicted (220 bpm – age); ≥9/10 on OMNI scale (pictorial scale for rating of perceived exertion ^7^); VO_2_ plateau based on visual analysis. Minute ventilation, peak O_2_ uptake and CO_2_ production results were averaged from the last 15 seconds of peak exercise. Maximum load, heart rate and respiratory rate were the highest recorded value at the peak of exercise. Ventilatory reserve was calculated by the following equation: 1-(minute ventilation/maximal voluntary ventilation)*100, where MVV = FEV_1_ x 35 ^8^. An automated volume calibration and gas analyser calibration were performed on each day of testing, in line with manufacturer’s instructions.


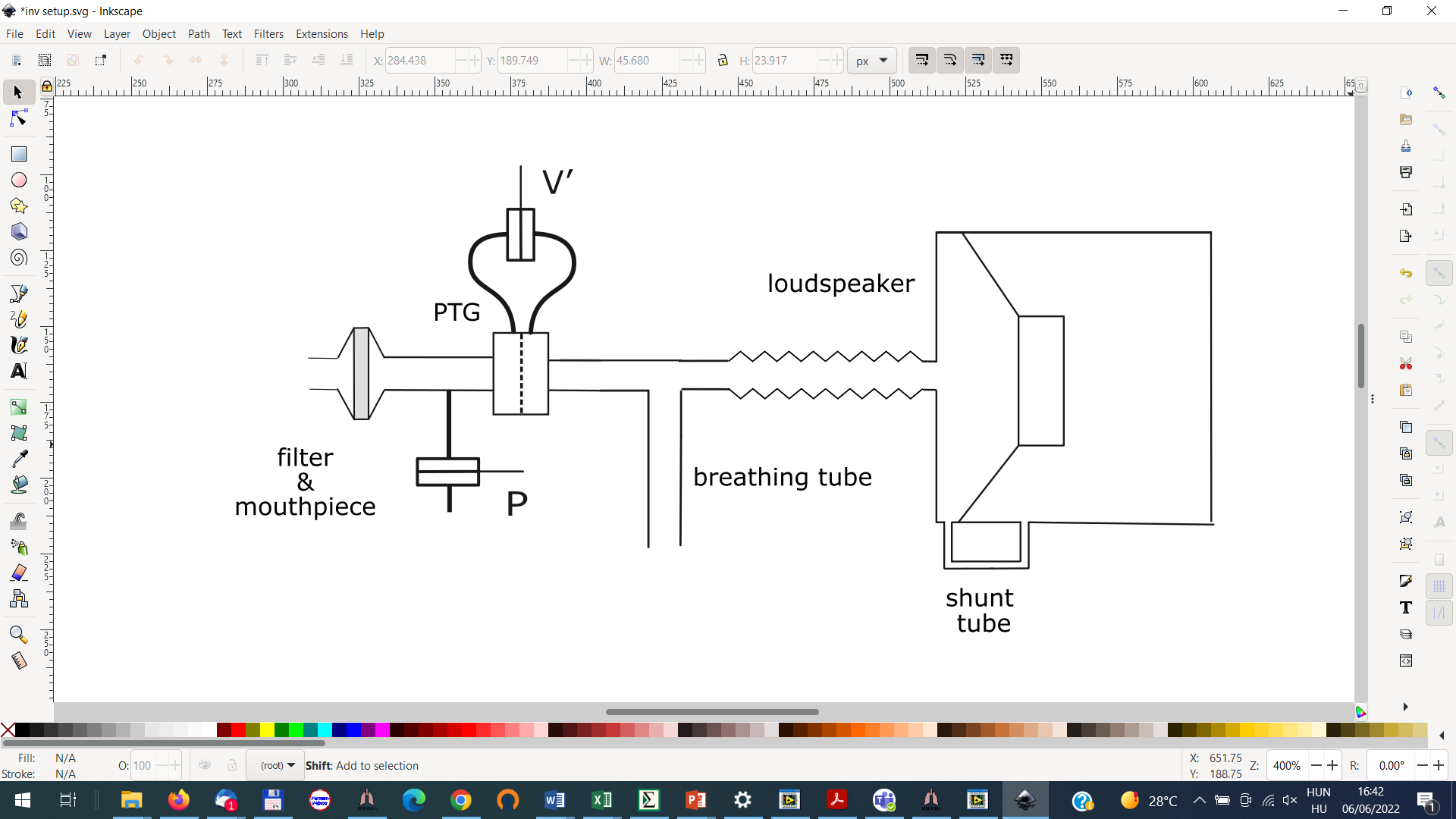


**Figure E1.** Schematic arrangement of the oscillometry device. PTG: pneumotachograph; pressure (P) and flow (V’) sensors: Honeywell model 26PCAFA6D (Golden Valley, MN, USA). Antibacterial filter with mouthpiece (Microgard-II microbial filter, Vyaire, Germany).

| **Group** | **Abbreviation** | **Definition** |
| --- | --- | --- |
| Prematurity-associated obstructive lung disease | POLD | FEV_1_ <LLN; FEV_1_/FVC ratio <LLN) |
| Prematurity-associated preserved ratio of impaired spirometry | pPRISm | (FEV_1_ <LLN; FEV_1_/FVC ratio ≥LLN) |
| Preterm Controls | PT_c_ | FEV_1_ ≥LLN |
| Term Controls | T_c_ | %FEV_1_ >90% |

**Table E1.** Abbreviations and definitions for grouping participants based on lung function.

|  | **POLD** | **pPRISm** | **1** | **2** | **T_c_** |
| --- | --- | --- | --- | --- | --- |
|  |  |  | **PT_c_ (Original)** | **PT_c_ (%FEV_1_ >90%)** |  |
| **n=** | 15 | 11 | 93 | 63 | 60 |
| **Rrs_mean_,**  **hPa.s/L** | 7.0 (6.0 to 8.0) | 6.4 (5.3 to 7.4) | 5.3 (5.1 to 5.6) | 5.2 (4.9 to 5.5) | 5.3 (5.0 to 5.7) |
| **Rrs_6_,**  **hPa.s/L** | 8.2 (7.0 to 9.4) | 7.0 (5.9 to 8.0) | 5.7 (5.3 to 6.0) | 5.5 (5.2 to 5.9) | 5.5 (5.1 to 6.0) |
| **Rrs_20_,**  **hPa.s/L** | 6.0 (5.2 to 6.8) | 6 (4.9 to 7.1) | 5.1 (4.9 to 5.3) | 5.1 (4.8 to 5.4) | 5.2 (4.9 to 5.5) |
| **Rrs_6-20_,**  **hPa.s/L** | 2.2 (1.5 to 2.9) | 1.0 (0.5 to 1.4) | 0.6 (0.4 to 0.8) | 0.4 (0.2 to 0.6) | 0.4 (0.2 to 0.6) |
| **Xrs_6_,**  **hPa.s/L** | -4.7 (-5.7 to -3.6) | -3.1 (-3.7 to -2.4) | -2.2 (-2.4 to -2.0) | -2.0 (-2.2 to -1.8) | -2.1 (-2.3 to -1.8) |
| **Xrs_20_,**  **hPa.s/L** | -1.9 (-2.7 to -1.2) | -1.0 (-1.4 to -0.5) | -0.3 (-0.5 to -0.2) | -0.2 (-0.4 to 0.0) | -0.2 (-0.4 to 0.0) |
| **Crs,**  **ml/hPa** | 5.4 (4.1 to 6.8) | 7.9 (6.3 to 9.5) | 11.3 (10.3 to 12.2) | 12.1 (10.9 to 13.2) | 11.9 (10.7 to 13.0) |
| **f*res*,**  **Hz** | 33.8 (28.9 to 38.7) | 26.6 (23.5 to 29.7) | 22.5 (21.1 to 23.9) | 21.4 (19.7 to 23.1) | 21.7 (20.0 to 23.4) |
| **AX,**  **hPa/L** | 62.6 (43.5 to 81.8) | 29.6 (19.3 to 39.9) | 19.1 (15.4 to 22.9) | 15.5 (12.0 to 19.0) | 16.1 (12.5 to 19.7) |

**Table E2.** Table showing sensitivity analysis of baseline data with preterm control (PT_c_) group in different formats, as 1) original grouping (FEV_1_ ≥LLN), and 2) only preterm children with %FEV_1_ >90% as per term controls.

**Abbreviations: POLD** – Prematurity-associated obstructive lung disease; **pPRISm** – prematurity-associated preserved ratio of impaired spirometry; **T_c_** – term control; **Rrs_mean_** – average respiratory system resistance 6-20 Hz; **R / Xrs_6 / 20_** – respiratory system resistance (Rrs) / reactance (Xrs) at 6 / 20 Hz; **Rrs_6-20_** – Frequency dependence of resistance between 6 – 20 Hz; **Crs** – compliance; ***ƒ*res** – resonant frequency; **AX** – area above reactance curve between 6 Hz and f*res*.

**References**

1. Edwards MO, Kotecha SJ, Lowe J, Richards L, Watkins WJ, Kotecha S. 2016. Management of prematurity-associated wheeze and its association with atopy. PLoS One. 11(5):e0155695.

2. Goulden N, Cousins M, Hart K, Jenkins A, Willets G, Yendle L, Doull I, Williams EM, Hoare Z, Kotecha S. 2021. Inhaled corticosteroids alone and in combination with long-acting β-2 receptor agonists to treat reduced lung function in preterm-born children; a randomized clinical trial. JAMA Pediatr. 176(2):133-141.

3. Cousins M, Hart K, Kotecha SJ, Henderson AJ, Watkins WJ, Bush A, Kotecha S. 2023. Characterising airway obstructive, dysanaptic and prism phenotypes of prematurity-associated lung disease. Thorax.Published Online First: 01 February 2023.

4. Shackleton C, Czovek D, Grimwood K, Ware RS, Radics B, Hantos Z, Sly PD. 2018. Defining ‘healthy’ in preschool-aged children for forced oscillation technique reference equations. Respirology. 23(4):406-413.

5. Miller MR, Hankinson J, Brusasco V, Burgos F, Casaburi R, Coates A, Crapo R, Enright P, van der Grinten CP, Gustafsson P et al. 2005. Standardisation of spirometry. Eur Respir J. 26(2):319–338.

6. Quanjer PH, Stanojevic S, Cole TJ, Baur X, Hall GL, Culver BH, Enright PL, Hankinson JL, Ip MS, Zheng J et al. 2012. Multi-ethnic reference values for spirometry for the 3-95-yr age range: The global lung function 2012 equations. Eur Respir J. 40(6):1324–1343.

7. Barkley JE, Roemmich JN. 2008. Validity of the CALER and OMNI-bike ratings of perceived exertion. Med Sci Sports Exerc. 40(4):760-766.

8. Joshi S, Powell T, Watkins WJ, Drayton M, Williams EM, Kotecha S. 2013. Exercise-induced bronchoconstriction in school-aged children who had chronic lung disease in infancy. J Pediatr. 162(4):813-818 e811.
